# Point prevalence and risk ractors of hospital acquired infections in a cluster of university affiliated hospitals in Shirz, Iran

**DOI:** 10.1186/1753-6561-5-S6-P237

**Published:** 2011-06-29

**Authors:** M Askarian, M Yadollahi, O Assadian

**Affiliations:** 1 Community Medicine, Shiraz University of Medical Sciences, Shiraz, Iran, Islamic Republic Of; 2 Department for Plasma Medicine, Ernst-Moritz-Arndt University Greifswald, Walther Rathenau Strasse 49a, 17489 Greifswald, Germany

## Introduction / objectives

Hospital acquired infections (HAIs) are one of the most critical complications in hospitalized patients, responsible for a major health and economic burden. The aim of this point prevalence study of HAI was conducted in Shiraz, Iran.

## Methods

The study was designed as four point prevalence surveys with identical design in eight university hospitals, each consisting of 60-700 beds, during all four season in 2008-2009. All patients admitted for ≥ 48 hours were studied. For all patients, a standardized data collecting form was completed, consisting of name, age, gender, presence or absence of HAI, administration of any antibiotic, insertion of central line, an endotracheal tube, mechanical ventilation, and any urinary catheter. HAI’s definitions were based on the US National Nosocomial Infection Surveillance (NNIS) definitions.

## Results

Data from 3450 patients were analyzed. The prevalence of HAI found to be 9.4%. The most common HAIs were blood stream infection (2.5%), surgical site infection (2.4%), urinary tract infection (1.4%), and pneumonia (1.3%). Logistic regression analysis showed that the OR of acquiring infections in males was 1.56 (95%CI 1.21-2.02), higher than in females. Other risk factors for HAI included central intravascular catheter adjusted OR 3.86 (95% CI 2.38-6.26), and urinary catheter adjusted OR 3.06 (95% CI 2.19-4.28).

## Conclusion

This point prevalence study showed that HAIsare frequent in Shiraz university hospitals, and that the proportion of antibiotic prescription is high. It implies more efforts in primary prevention of HAI associated with the use of indwelling devices, and prevention of SSI.

## Disclosure of interest

None declared.

